# Does Western or Chinese Zodiac Sign Predict COVID Infections and Death?

**DOI:** 10.7759/cureus.56165

**Published:** 2024-03-14

**Authors:** Amanda Frugoli, Sagar Parekh, Graal Diaz

**Affiliations:** 1 Graduate Medical Education, Internal Medicine, Community Memorial Hospital, Ventura, USA; 2 Graduate Medical Education, Community Memorial Hospital, Ventura, USA; 3 Graduate Medical Education, Community Memorial Health System, Ventura, USA

**Keywords:** covid, sars-cov2, western zodiac, horoscope, covid-19, astrology, chinese zodiac

## Abstract

Introduction: Humans have been fascinated by and studying the sky since the beginning of time. Beliefs in Chinese and Western astrology persist in modern society and have gained increasing interest in light of the COVID-19 pandemic. Zodiac signs are typified by certain qualities, for example, obsessive-compulsive traits in Libras and Virgos or the highly social traits in Leos and Geminis. We investigate whether the various characteristics or personalities purported of assigned birth signs may alter the predisposition to COVID-19 infections or mortality.

Methods: This is a retrospective, single-center cohort study of 2545 adult patients with confirmed COVID-19 infection presenting to the emergency room over a 14-month period (September 2020 to November 2021). COVID-19 infectivity was determined based on polymerase chain reaction (PCR) testing. Western and Chinese Zodiac signs were designated using date of birth. Both Zodiac signs were evaluated for risk of infection and death.

Results: Mortality rates across the zodiac and astrology signs showed no statistical difference using the 12-sample test for equality of proportions. Coincidentally, the mean age for the deceased was 74.5 years, and it was 53.9 years for those alive, resulting in a difference of 20.6 years. A two-sample t-test confirms that the observed difference of 20.6 years of age between the two groups is statistically significant with a p-value <0.05. The coefficient of the predictor age is statistically significant. The odds ratio estimate of age is 1.06, with the corresponding 95% confidence interval (CI) being (1.048, 1.073). This means that the odds of dying increase by 6% for every additional year.

Discussion: Astrology once held a significant impact on beliefs in medicine and continues in Chinese and Ayurvedic medicine. Our study utilized local data to determine if COVID-19 infection rates and mortality might have a relationship to astrological designations of Chinese and Western zodiac signs. Data analysis demonstrated that there was no statistical significance found between Western and Chinese Zodiac signs and mortality or infections. Similar to many previous studies, age can be a risk factor for mortality.

## Introduction

Humans have been fascinated by and studying the sky since the beginning of time. Astrology was once looked to in the prediction of illness and death of princes, kings, and popes during the Italian Renaissance. Astrologers and their prognostications were a privileged source of information during these times [1]. Furthermore, several British practitioners amassed statistical evidence which purported to prove the influence of the Moon on fevers and other diseases. Some, like James Lind, were widely respected and drew support for their views from such influential figures as English physician Erasmus Darwin, the grandfather of Charles Darwin [2]. Two decades ago, Welham et al. completed a meta-analysis that demonstrated increased schizophrenia in babies born during winter and spring, correlating to winter zodiacs of Aquarius, Capricorn, and Sagittarius; and spring zodiacs of Taurus, Aries, and Pisces [3].

**Figure 1:**
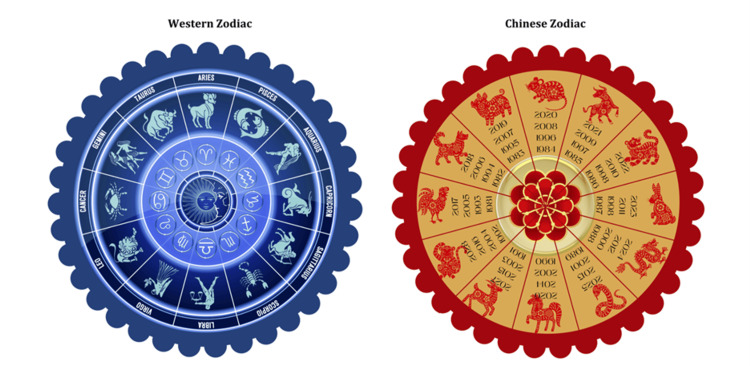
Zodiacs This image was created by the authors using Canva.

The 12 Zodiac signs or constellations are associated with astrological principles that the positions of stars and planets can affect human lives, personalities, and behaviors [4]. The Zodiac houses are divided into 12 signs based on birth month. The Zodiac houses or signs include Aries the Ram, Taurus the Bull, Gemini the Twins, Cancer the Crab, Leo the Lion, Virgo the Virgin, Libra the Balance, Scorpio the Scorpion, Sagittarius the Archer, Capricornus the Goat, Aquarius the Water Bearer, and Pisces the Fish (Figure 1). In contrast, the Chinese calendar also has a classification scheme to divide attributes to animal signs based on a 12-year cycle. This cycle is not based on an associated constellation but spans a 12-part recurring cycle by years (Figure 1).

In contemporary society, particularly those of Indian and Chinese culture, there remains a firm belief in astrological principles and their impact on human health. It is believed that highly advanced knowledge related to astrology on medicine is preserved in Indian scripture and this knowledge was transmitted from generation to generation. These scriptures are utilized as a means for health promotion, prevention, and cure. The Ayurvedic physicians, in conjunction with the astrologer and Hindu priest, would simultaneously look after the health and welfare of individuals, families, and the country [5]. The strong Hindu embrace of astrology can even lead to a desire to control their exact time of death [6].

Similarly, Chinese culture embraces the Chinese zodiac with influence over medical decision-making. It has been found that the preference for childbirth during the Dragon Year has influenced the fertility of modern Chinese populations through zodiacal birth-timing motivations [7]. There also is a presence of discrimination and stereotyping that are brought along in Chinese culture from the translation of astrological signs from Western culture. For instance, Virgos are stereotyped as having disagreeable personalities, likely because of Virgo’s Chinese translation as "virgin." With these stereotypes, Chinese individuals were found to discriminate against Virgos in romantic dating as well as in job recruitment [8].

Many Americans and other nations share superstitions regarding how astrology influences our personality dispositions. Ancient Greek cultures, fortune tellers, and magazines in horoscope readings proposed that constellations and planetary alignments have predictive properties specified toward zodiac signs. Amid the COVID-19 pandemic, there has been an upsurge in the utilization of the Chinese and Western Zodiac, likely related to the uncertain conditions worldwide [9]. In a recent evaluation of vaccination rates, the Salt Lake City Health Department identified different vaccination rates based on Zodiac signs [10]. In this study, we investigate if the Chinese or Western Zodiac signs have a predisposition to COVID-19 infections or mortality. This research was presented as a meeting abstract at the American College of Osteopathic Internists (ACOI) Annual Scientific Meeting in Tampa, Florida, on October 14, 2023.

## Materials and methods

This is a retrospective, single-center cohort study of 2545 adult patients with laboratory-confirmed COVID-19 presenting to our emergency room over 14 months (September 2020 to November 2021). Laboratory COVID infection was determined by respiratory PCR testing. Western and Chinese Zodiac signs were determined using date of birth, with the Chinese Zodiac determined based on birth year and the Western Zodiac determined using birth month. This is detailed in Table 1 and illustrated in Figure 1.

**Table 1:** Zodiac Sign Determination Based on the Date of Birth

Western Zodiac	Chinese Zodiac
Aries (March 21–April 19)	Dog (1922, 1934, 1946, 1958, 1970, 1982, 1994, 2006, 2018)
Taurus (April 20–May 20)	Pig (1923, 1935, 1947, 1959, 1971, 1983, 1995, 2007, 2019)
Gemini (May 21–June 21)	Rat (1912, 1924, 1936, 1948, 1960, 1972, 1984, 1996, 2008, 2020)
Cancer (June 22– July 22)	Ox (1913, 1925, 1937, 1949, 1961, 1973, 1985, 1997, 2009, 2009, 2021)
Leo (July 23–August 22)	Tiger (1914, 1926, 1938, 1950, 1962, 1974, 1986, 1998, 2010, 2022)
Virgo (August 23–September 22)	Rabbit (1915, 1927, 1939, 1951, 1963, 1975, 1987, 1999, 2011, 2023)
Libra (September 23– October 23)	Dragon (1916, 1928, 1940, 1952, 1964, 1976, 1988, 2000, 2021, 2024)
Scorpio (October 24– November 21)	Snake (1917, 1929, 1941, 1953, 1965, 1977, 1989, 2001, 2013, 2025)
Sagittarius (November 22– December 21)	Horse (1918, 1930, 1942, 1954, 1966, 1978, 1990, 2002, 2014, 2026)
Capricorn (December 22– January 19)	Sheep (1919, 1931, 1943, 1955, 1967, 1979, 1991, 2003, 2015, 2027)
Aquarius (January 20– February 18)	Monkey (1920, 1932, 1944, 1956, 1968, 1980, 1992, 2004, 2016, 2028)
Pisces (February 19– March 20)	Rooster (1921, 1933, 1945, 1957, 1969, 1981, 1993, 2005, 2017, 2029)

Statistical methods

Due to the retrospective nature, a sample size of convenience was utilized. The distribution of Zodiac signs was determined with percentage. A 12-sample test for equality of proportions was conducted. The test's null hypothesis was that all 12 proportions were equal, and the corresponding alternative hypothesis was that at least one of the 12 proportions was different.

Both Zodiac signs were evaluated for risk of infection and risk of death using the 12-sample test for equality of proportions. A two-sample t-test evaluated the age between the two groups. Next, the population was divided using quartiles, and four age groups were created: 18-38, 39-55, 56-71, and >71 and stratified into boxplots, to explore whether there is a linear trend in the proportion of deaths across ages. A chi-squared test for a trend in the proportions of deaths was completed with follow-up linear regression to estimate the impact of age on the death rate. The Institutional Review Board approval was obtained. 

## Results

There were 2545 patients identified and included for analysis. Table 2 outlines patient demographics and the distribution of Zodiac signs. The percentage of Zodiac signs appears to be uniform across each respective Chinese Zodiac sign, where Rooster had the fewest representation (7.4%) of the sample, and Rabbit had the highest (9.1%). Similarly, the astrology signs were uniformly represented across the entire cohort, with Scorpio having the fewest at 6.7% and Leo as the largest subgroup at 9.4%. A 12-sample test for equality of proportions was conducted.

**Table 2:** Summary Statistics

Demographic Variables	Count	% of Population	P-Values	% of Death	P-Values
Sex					
Male	1235	48.50%	NS		
Female	1310	51.50%			
Asian	62	2.40%			
Black/African American	65	2.60%			
Native Hawaiian or Pacific Islander	5	0.20%			
Latin X or Unknown	1278	50.20%			
White	1135	44.60%			
Western Zodiac					NS
Aquarius	219	8.61%		3.65%	
Aries	199	7.82%		5.53%	
Cancer	201	7.90%		2.49%	
Capricorn	213	8.37%		3.76%	
Gemini	212	8.33%		4.72%	
Leo	239	9.39%	NS	4.18%	
Libra	222	8.72%		7.21%	
Pisces	217	8.53%		3.23%	
Sagittarius	223	8.76%		4.93%	
Scorpio	171	6.72%		5.26%	
Taurus	216	8.49%		6.02%	
Virgo	213	8.37%		5.16%	
Chinese Zodiac					NS
Dog	209	8.21%		6.22%	
Dragon	196	7.70%		5.61%	
Horse	218	8.57%		3.21%	
Monkey	215	8.45%		3.26%	
Ox	202	7.94%		5.45%	
Pig	193	7.58%		4.66%	
Rabbit	232	9.12%		3.45%	
Rat	230	9.04%	NS	5.65%	
Rooster	187	7.35%		3.21%	
Sheep	216	8.49%		4.63%	
Snake	222	8.72%		6.76%	
Tiger	225	8.84%		4.00%	

The test's null hypothesis was that all 12 proportions were equal, and the corresponding alternative hypothesis was that at least one of the 12 proportions was different. The test statistic had a p-value that was nonsignificant and greater than the statistically significant threshold of 0.05. Thus, no statistically significant difference was established in the rates of infections among the various zodiac designations.

**Figure 2:**
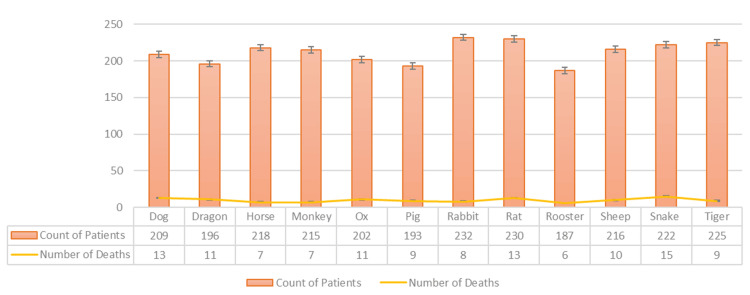
COVID-Related Hospitalization and Death: By Chinese Zodiac The test's null hypothesis was that all 12 proportions were equal, and the corresponding alternative hypothesis was that at least one of the 12 proportions was different.

**Figure 3:**
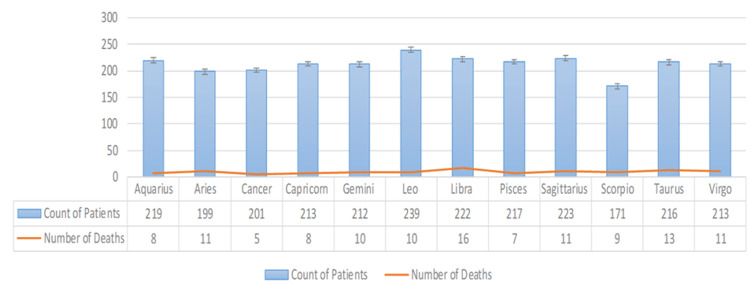
COVID-Related Hospitalization and Death: By Western Zodiac The test's null hypothesis was that all 12 proportions were equal, and the corresponding alternative hypothesis was that at least one of the 12 proportions was different.

There were 119 (4.6%) deceased patients in this dataset (Figures 2, 3). Mortality rates across the zodiac and astrology signs showed no statistical difference using the 12-sample test for equality of proportions.

**Figure 4:**
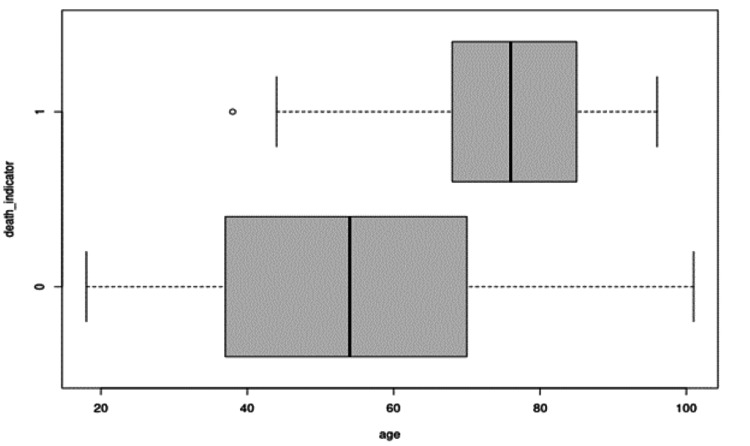
Boxplot of Ages Between Deceased and Alive Patients

Figure 4 displays a boxplot of ages between those who died and those who were alive. Coincidentally, the mean age for the deceased was 74.5 years, and it was 53.9 years for those alive, resulting in a difference of 20.6 years. A two-sample t-test confirms that the observed difference of 20.6 years of age between the two groups is statistically significant with a p-value <0.05. Based on the overlap of boxplots, we can explore whether there is a linear trend in the proportion of deaths across ages. The population was divided using quartiles, and four age groups were created: 18-38, 39-55, 56-71, and >71. There appears to be a trend in the proportion of deaths with an increase in age among the entire cohort.

Conducting a chi-squared test for a trend in the proportions of deaths results in a p-value <0.05. We can estimate the impact of age on the death rate by performing a logistic regression. Figure 4 shows that the coefficient of the predictor age is statistically significant. The odds ratio estimate of age is 1.06, with the corresponding 95% confidence interval (CI) being (1.048, 1.073). This means that the odds of dying increase by 6% for every additional year of age.

## Discussion

Historically, astrology and zodiac signs are entities with a shared global interest. We hypothesized that there may be differences in mortality and infection rates associated with COVID-19 depending on a patient's astrological sign. The hypothesis was chosen in light of the various designations of personality traits associated with the different zodiac signs. Furthermore, there remains a large preponderance in our society, as well as in Indian and Chinese communities, on the reality of astrological influence on our actions, personalities, and health.

In this retrospective study, we utilized a dataset of 2545 patients over the course of one year during the height of the COVID-19 pandemic. Analysis was performed with a 12-sample test for equality of proportions to explore the various astrological designations of individuals and describe if there may be a variation in COVID-19 mortality. Analysis of data in our study demonstrated that there was no statistical significance found between Western and Chinese Zodiac signs in mortality or infection rates. We are unable to support an association between astrological signs and the risk of COVID-19 infection or mortality. There is ongoing interest in this correlation, and Morgan et al. were able to identify an association of Western Zodiac signs with medical specialty [11]. Our findings are more in line with prior handfuls of negative studies that have failed to find an association between astrology and personality traits [12].

It is also possible that the risk of infection and correlation with the Zodiac sign diminished with time as the prevalence of infection increased. A national review of patients with infections within the first three months may have had different outcomes, but due to our data and methods, this could not be determined.

The logistic regression demonstrates increased mortality with increased age. It is estimated a 6% increased chance of dying with each additional year of life. This is consistent with details already well-researched in COVID-19 [13,14,15]. COVID-related mortality has been shown to increase exponentially with older age, and after age 75, even without comorbid conditions. The finding of older age and mortality is thought to be due to less robust immune responses.

Albeit the amount of evidence against the influence of Chinese and Western Zodiac signs seen in the literature, there remains a presence of these superstitious beliefs worldwide and ongoing investigations into their possible meaning. It remains valuable to demonstrate the invalidity of such beliefs, as was displayed with the results of this study. Nonetheless, there is much in science and medicine that remains unknown.

Limitations

Due to the retrospective nature of this study design, we were unable to control for confounding variables, including vaccination status, medical treatments, and existing comorbidities. 

## Conclusions

Astrology has a long history in medicine and continues in Chinese and Ayurvedic practice. Our retrospective study investigating Western Zodiac and Chinese Zodiac signs in COVID hospitalization and mortality was unable to support an astrological correlation. The research is consistent with prior studies demonstrating age is a risk factor for COVID-related mortality, with an estimated 6% increased risk of dying with each additional year of life. 
